# Measurement method research of Chinese texts’ difficulty based on two-characters continuations

**DOI:** 10.1371/journal.pone.0309717

**Published:** 2024-09-04

**Authors:** Dongjie Zhou, Tianqing Zheng

**Affiliations:** 1 School of Humanities, Fujian University of Technology, Fuzhou, Fujian, China; 2 Fujian Key Laboratory of Intelligent Machining Technology and Equipment, Fujian University of Technology, Fuzhou, Fujian, China; Federal University of Paraiba, BRAZIL

## Abstract

Two-characters continuation, which is a string with two characters emerging in linear sequence, can break through the encapsulation and independence of long solidified language chunks (words and phrases). In this way, two-characters continuation can measure the information of not only static language units (words and phrases) but also their combination in the text. Therefore, two-characters continuation is used as a measurement unit for investigating Chinese text’s difficulty, to enhance the accuracy of measuring text’s difficulty. Three different measurement methods of text’s difficulty are proposed, which are respectively based on "continuation index of character", "new and stable two-characters continuation" and "emerging tendency of two-characters continuation". The results show that compared to other two methods, the measurement method of text’s difficulty based on new and stable two-characters continuations has better effectiveness, whose accuracies for measuring text’s difficulty with 6 levels, 3 levels and 2 levels difficulties can reach 36.4%, 64.6% and 79.6%, respectively. In addition, compared to Jiang and Wu’s research works, the above measurement method also shows a better effectiveness.

## Introduction

Learning efficiency of texts can be effectively enhanced by reading texts with appropriate difficulty [1–3]. It is an important research topic to control the difficulty of students’ learning and reading materials in the field of basic education and the second language teaching [4–8]. Especially in recent years, the research fields, such as the evaluation of composition’s difficulty, the publication of children’s book, the recommendation of extracurricular reading material and the personalized retrieval, all involve the quantification of the difficulty of Chinese text, which need higher requirements for the quantification of text’s difficulty.

There are many research works on the measurement of text’s difficulty in and outside China [9–12]. For English texts, related researches could be traced back to the 1920s [13]. As for Chinese texts, researches on the quantification of text’s difficulty have fallen behind. The first research work on the measurement of texts’ difficulty did not appear until 1971 [14].

At present, the main methods of measurement of text’s difficulty are statistical means. Jiang predicted the text’s difficulty of Chinese compositions in primary school based on convolutional neural network model [15]. Wu et al. established a language feature system to measure the text’s difficulty of Chinese textbooks in primary school using support vector machine model [16]. Schwarm et al. used support vector machine model to predict the difficulty of English text [17]. Mcnamara et al. used text quantitative analysis tool based on Coh-Metrix to analyze the readability of text [18]. However, the above measurement systems are complex, which often yield unsatisfactory results in measuring the difficulty of text [19,20]. The current state of researches on the measurement of text’s difficulty in China fails to meet the demand of market. Therefore, it is urgent and necessary to further strengthen the measurement of Chinese text’s difficulty.

To explore a more simple and effective method for measuring the difficulty of Chinese text, Chinese-character, which is as a natural and explicit unit in text, is investigated in this study. Monosyllabic morphemes, which account for more than 93% of Chinese morphemes are represented by a single character in writing. Character is the most basic recording and describing unit of Chinese, and it is the central theme of Chinese, the intersection of pronunciation, semantics, grammar and vocabulary, and the foundation of Chinese, which is closely related to Chinese [21]. Moreover, excepting for polysyllabic words, the words and many solidified and temporary word statement-chunks are assembled by choosing characters. In this way, the combination of the character and the character, namely the two-characters continuation, can break through the encapsulation and independence of long solidified language chunks (words and phrases), to measure the information of static language units and their combination in the text. Therefore, two-characters continuation is used as research object in the measurement of text’s difficulty.

In this study, a hierarchical corpus of primary school students’ compositions with 2.8 million characters is constructed, which is divided into training corpus and testing corpus with a ratio of 9:1. The source of the compositions is from eight journals, which are *Composition for Primary School Students*, *Excellent Composition for Primary School Students*, *Story Composition*, *Innovative Composition*, *Happy Composition*, *Composition and Examination*, *New Composition*, *and Colorful Chinese*. The training corpus and the multiple statistical methods are used to obtain the resource corpuses of two-characters continuations with different difficulty levels. Then, according to the characteristics of the different resource corpuses, the corresponding algorithms for measuring text’s difficulty are designed. Finally, the better method of measuring text’s difficulty is determined by comparing the results obtained from different measurement methods.

## Method

### Method for measuring text’s difficulty based on continuation index of character

#### Application value of continuation index of character in measuring text’s difficulty

The difficulties of characters used in a text is an important indicator of the overall difficulty level of a text. The difficulty of a character can be reflected by the number of kinds of adjacent coexisting characters in training corpus. If a character can coexist with a larger number of other characters, it indicates that this character is familiar to people, implying that the difficulty of this character is low. The continuation index of a character reflects the number of characters that can appear adjacent to the character in training corpus, which can be determined by calculating the number of two-characters containing this character. Based on the above analysis, the continuation index of character can be used to represent its difficulty, measuring the difficulty of the text.

#### Method introduction of continuation index of character

Calculation equation of the average of continuation indexes of the characters in testing text is shown in Eq (1).


$$X_{t}=\frac{\sum_{k=1}^{m}C_{k}}{m}$$ (1)

*X_t_* represents the average of continuation indexes of the characters in testing text. *C_k_* represents the number of kinds of two-characters continuations containing the character *k*. *m* represents the number of kinds of characters in testing text.

Absolute distance method indicates the absolute distance between two points in one dimensional space. The difficulty level of testing text is measured by calculating the absolute distance between the average of continuation indexes of the characters in testing text and that of all texts with the same difficulty level in training corpus. Calculation equation of absolute distance is shown in Eq (2).


$$Z=|X_{t}-\bar{X_{i}}|$$ (2)

In Eq (2), *Z* represents the absolute distance between the average of continuation indexes of the characters in testing text and that of all texts with the same difficulty level in training corpus. *X_t_* represents the average of continuation indexes of the characters in testing text. $\bar{X_{i}}$ represents the average of continuation indexes of the characters of all texts with the difficulty level *i* in training corpus.

**Fig 1:**
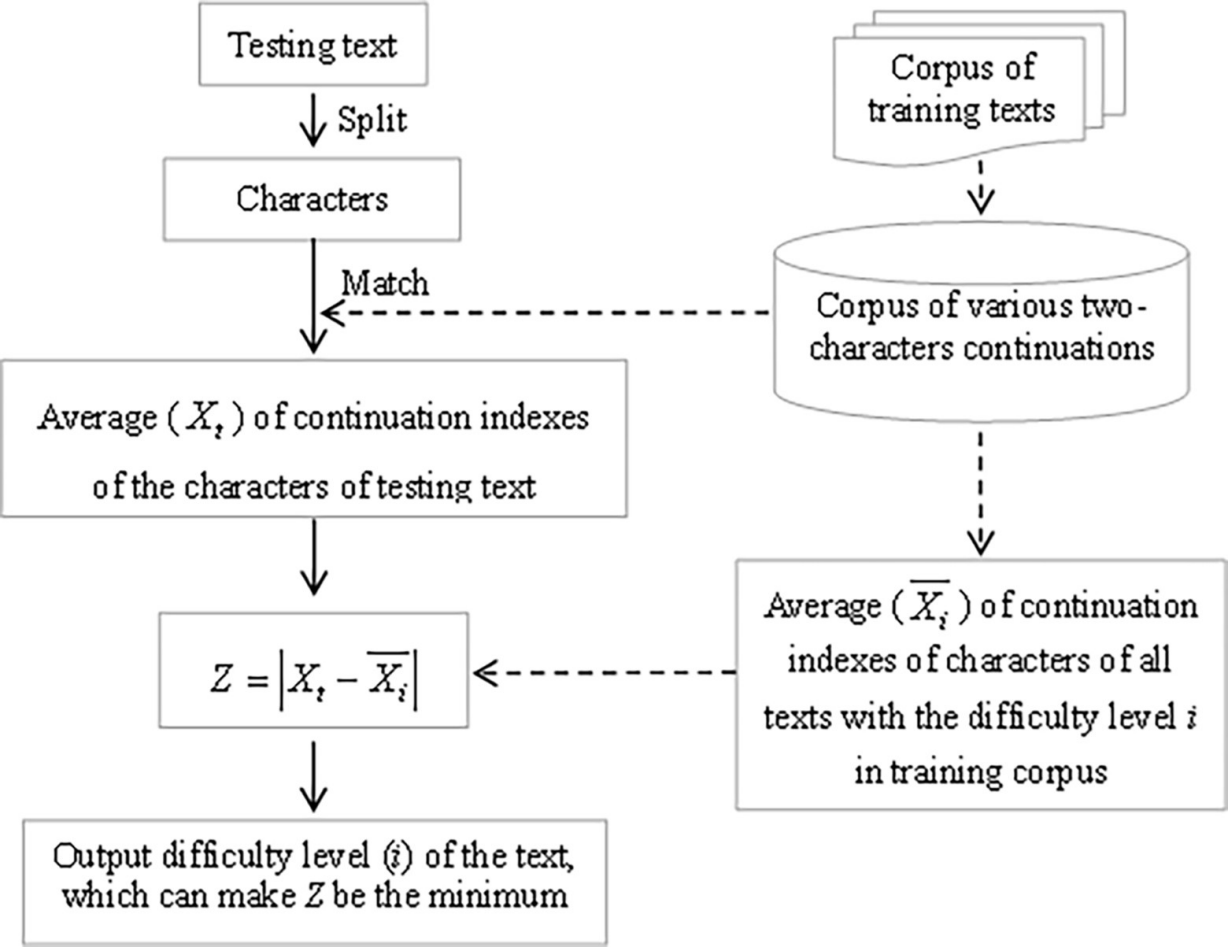
Working principle for measuring text’s difficulty based on continuation index of character.

The process for measuring text’s difficulty based on resource corpus of continuation index of character and absolute distance method can be seen in Fig 1. Measurement of the text’s difficulty with 6 levels difficulty is used as an example, whose process is as follow. The average of continuation indexes of the characters in testing text is regarded as the value (*X_t_*) of a point in Eq (2). The average of continuation indexes of the characters of all texts with the *i*-th level of difficulty in training corpus is regarded as the value ($\bar{X_{i}}$) of the other point in Eq (2). The point "the testing text" is closest to the point "all texts with the same level of difficulty", indicating that the difficulty of the testing text is tendency to be this specific level. Tables 1–3 show averages of continuation indexes of characters of same level texts with 6 levels, 3 levels and 2 levels difficulty, respectively in training corpus.

**Table 1:** Average of continuation indexes of characters of same level texts with 6 levels difficulty in training corpus.

6 levels	Average of continuation indexes of characters
The 1st level (language level of 6 year old children)	881.125
The 2nd level (language level of 7 year old children)	838.770
The 3rd level (language level of 8 year old children)	733.481
The 4th level (language level of 9 year old children)	674.700
The 5th level (language level of 10 year old children)	651.560
The 6th level (language level of 11 year old children)	643.023

**Table 2:** Average of continuation indexes of characters of same level texts with 3 levels difficulty in training corpus.

3 levels	Average of continuation indexes of characters
The 1st level (language level of 6–7 year old children)	848.118
The 2nd level (language level of 8–9 year old children)	707.763
The 3rd level (language level of 10–11 year old children)	647.743

**Table 3:** Average of continuation indexes of characters of same level texts with 2 levels difficulty in training corpus.

2 levels	Average of continuation indexes of characters
The 1st level (language level of 6–8 year old children)	797.860
The 2nd level (language level of 9–11 year old children)	656.438

### Method for measuring text’s difficulty based on new and stable two-characters continuation

#### Application value of new and stable two-characters continuation in measuring text’s difficulty

Since the difficulty of primary school students’ composition is increasing with the increase of the student’s grade, the usage of two-characters continuations in the texts is changed with the difficulty levels of the texts. Moreover, some stable two-characters continuations exist in the texts with a specific level of difficulty, and they are the representative of the difficulty of the texts and the key to measure the difficulty of the testing text. By extracting stable continuations from the texts with a specific level of difficulty in the training corpus, the corpus of new and stable two-characters continuations with different levels of difficulty is obtained. According to the characteristic of the corpus, the corresponding measurement algorithm of text’s difficulty is designed to analyze the usage of two-characters continuations with different levels of difficulty in testing text and measure its level of difficulty.

#### Method introduction of new and stable two-characters continuation

The process for determining new and stable two-characters continuations for the texts with a specific level of difficulty in training corpus is as follows.

1. In the texts with the 6 levels, 3 levels and 2 levels difficulties, the criterion of thresholds of the frequency and the text distribution’s number for new and stable continuations of the texts with a specific level of difficulty, are shown in Table 4. When the frequency and the text distribution’s number of a continuation are set as 3 and 3, respectively, the extracted continuations not only have a certain scale but also appear in more texts. If the frequency and the text distribution’s number of some continuations in the texts with the 1st level of difficulty meet or exceed the correspondent thresholds, respectively, the above continuations belong to the new and stable continuations of the texts with the 1st level of difficulty.
2. If the frequency and the text distribution’s number of some continuations in the texts with the 2nd level of difficulty meet or exceed the corresponding thresholds, respectively, those continuations, which include those mentioned earlier but excluding new and stable continuations with the 1st level of difficulty, become the new and stable continuations with the 2nd level of difficulty.
3. If the frequency and the text distribution’s number of some continuations in the texts with the 3rd level of difficulty meet or exceed the correspondent thresholds, respectively, those continuations, which include the above continuations but excluding new and stable continuations with the 1st and 2nd level of difficulty are the new and stable continuations with the 3rd level of difficulty. Furthermore, the new and stable continuations of the texts with levels 4–6 difficulties, respectively, can be obtained.

**Table 4:** Thresholds of frequency and text distribution’s number of new and stable two-characters continuations.

Threshold Kind of difficulty level	Threshold of frequency	Threshold of text distribution’s number
Texts with 6 levels difficulty	3	3
Texts with 3 levels difficulty	3	3
Texts with 2 levels difficulty	3	3

According to Piaget’s theory of the stage development of child’s intelligence [22–24], the children from 7 to 12 years old have a fixed sequence of intelligence development. The sequence of intelligence development can not be reversed or spanned. Based on the above theory, it can be inferred that the composition texts in primary school become difficult with children’s age. Therefore, according to the characteristic of the composition texts in primary school and the input hypothesis of *i*+1 language of Krashe [25,26], *i*+1 measurement algorithm of language’s difficulty is proposed. In the algorithm, *i* represents the number of low-difficulty continuations used in a text. "1" is an abstract symbol which represents the number of high-difficulty continuations. When the number of the continuations with high difficulty in a text reaches a certain value, the overall difficulty level of the text is determined to be *i*+1, indicating high difficulty, otherwise indicating low difficulty. The difficulty of the texts is not limited to two levels.

**Fig 2:**
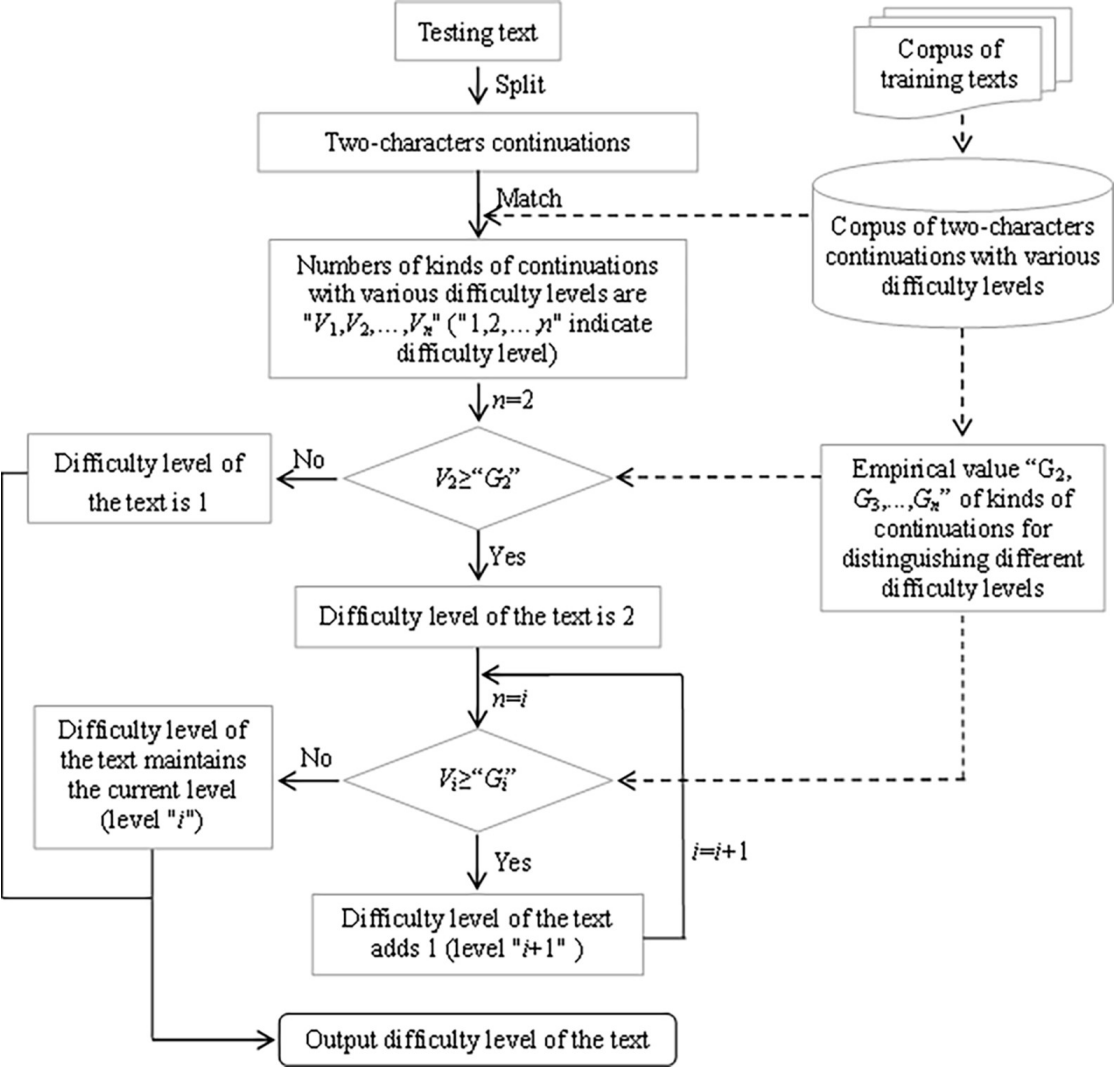
Working principle for measuring text’s difficulty based on *i*+1 algorithm.

The working principle of *i*+1 measurement algorithm of language’s difficulty is as follow, which can be seen in Fig 2. Different continuations corresponding to different levels of difficulty exist in testing text, that is, there are multiple different "1" (*V*_2_, *V*_3_,…,*V*_*n*_) in the text. When the number (*V*_2_) of kinds of continuations with the 2nd level of difficulty exceeds a empirical value *G*_2_, the difficulty of the text is “*i*”+1 (*i* represents the continuations with the most basic difficulty), otherwise the difficulty is "*i*". When the number (*V*_3_) of kinds of continuations with the 3rd level of difficulty exceeds a empirical value *G*_3_, the text’s difficulty increases one level, otherwise the difficulty remains the previous level. Until the number of kinds of continuations with the higher level of difficulty can not reach a certain value, the judgment of the text’s difficulty stops.

The core of the *i*+1 measurement algorithm of language’s difficulty is the setting of “1” in *i*+1. The values of “1” of the continuations with different levels of difficulty in training corpus directly affect the effectiveness of the algorithm. In fact, the value of “1” can not be determined subjectively, which must be determined according to the specific numbers corresponding to the usage of continuations with various levels of difficulty in the texts of training corpus. In addition, the value of “1” for the continuations with a specific level of difficulty must have the ability for effectively distinguishing the difference between the texts with the specific level of difficulty and its adjacency. Taking the measurement of text’s difficulty with 6 levels difficulty as an example, the method for determining the empirical values of “1” is explained in detail as below.

1. Technical route for determining the empirical value of “1” is introduced. The number of level 6 texts in training corpus is set to be *D*. The numbers of kinds of level 6 continuations of all level 6 texts in training corpus are counted, which are *S*_6,1_, *S*_6,2_, *S*_6,3_, …, *S*_6,*D*_. *A* is the minimum value in the *S*_6,1_, *S*_6,2_, *S*_6,3_, …, *S*_6,*D*_. The number of the level 5 texts in training corpus is set to be *E*. The numbers of kinds of level 6 continuations of all level 5 texts in training corpus are counted, which are *S*_5,1_, *S*_*5*,2_, *S*_5,3_, …, *S*_5,*E*_. *B* is the maximum value in the *S*_5,1_, *S*_*5*,2_, *S*_5,3_, …, *S*_5,*E*_.
  A variable *H* is set to be *A* at the very beginning. The number of the values in the *S*_6,1_, *S*_6,2_, *S*_6,3_, …, *S*_6,*D*_, which surpass *H*, is *d*. The number of the values in the *S*_5,1_, *S*_*5*,2_, *S*_5,3_, …, *S*_5,*E*_, which surpass *H*, is *e*. The *z* is set to be (*d*/*D*-*e*/*E*). The goal of this research is to find a specific value of the variable *H*, which can make *z* be the maximum by making *H* = *H*+1 at each stage, maximizing the distinguishability between level 6 and level 5 texts.
2. The process for determining the empirical value of “1” is introduced. The self-developed software is used to obtain the different values of *z* corresponding to different values of *H*, which are *Z*_1_, *Z*_2_, *Z*_3_, …, *Z*_*B-A*_. The value of *H*, which can obtain the maximum of the values of *Z*_1_, *Z*_2_, *Z*_3_, …, *Z*_*B-A*_, is the empirical value of “1”. If the value of *A* exceeds *B* at the beginning of the solving process, the empirical value of “1” is set to be *A*.
3. The obtained result is introduced, which can be seen in the Tables 5–7. The empirical value of “1” that can determine the text with the 6^th^ level of difficulty is gained, and then the empirical value of “1” that can determine the text with the 5^th^ level of difficulty is calculated. In this way, the empirical values of “1” that can determine the text with the levels 2–4 of difficulties, respectively, are also calculated.

**Table 5:** Empirical values of "1" for measuring the difficulty of the texts with 6 levels difficulty in training corpus.

Level Type	The 2nd level (*d*/*D*, *e*/*E*)	The 3rd level (*d*/*D*, *e*/*E*)	The 4th level (*d*/*D*, *e*/*E*)	The 5th level (*d*/*D*, *e*/*E*)	The 6th level (*d*/*D*, *e*/*E*)
Value of “1”	35 (0.67, 0.20)	20 (0.86, 0.06)	14 (0.92, 0.05)	15 (0.90, 0.08)	8 (0.89, 0.06)

**Table 6:** Empirical values of "1" for measuring the difficulty of the texts with 3 levels difficulty in training corpus.

Level Type	The 2nd level (*d*/*D*, *e*/*E*)	The 3rd level (*d*/*D*, *e*/*E*)
Value of “1”	30 (0.88, 0.07)	17 (0.92, 0.07)

**Table 7:** Empirical values of "1" for measuring the difficulty of the texts with 2 levels difficulty in training corpus.

Level Type	The 2nd level (*d*/*D*, *e*/*E*)
Value of “1”	27 (0.92, 0.05)

### Method for measuring text’s difficulty based on emerging tendency of two-characters continuation

#### Application value of emerging tendency of two-characters continuation in measuring text’s difficulty

The usage of a two-characters continuation in the texts with different levels of difficulty are different. The number of a two-characters continuation emerged in the texts with certain level of difficulty is counted, which is *O*. The number of the two-characters continuation emerged in the texts of all levels of difficulty is counted, which is *P*. The emerging tendency of two-character continuations in the text with specific level of difficulty is *O*/*P*. The difficulty level of a testing text can be determined by comparing the accumulation values of the tendencies of all two-characters continuations of the testing text in the training texts respectively with different levels of difficulty. Unlike classifying the difficulty of a two-character continuation in a specific level, the emerging tendency of the continuation serves as an indicator of the continuation’s difficulty, encompassing its occurrence across different levels of difficulty. This provides a comprehensive reflection of the continuation’s usage information, thereby enhancing its value in analysis of text’s difficulty.

#### Method introduction of emerging tendency of two-characters continuation

Calculation equations of the emerging tendency of continuation are shown in Eqs 3 and 4.


$$Q(i,j)=\frac{U_{i,j}}{\sum_{i=1}^{n}U_{i,j}}$$ (3)

$$\sum_{i=1}^{n}Q(i,j)=1$$ (4)

*Q*(*i,j*) represents the emerging tendency of continuation *j* in the training texts with the *i-th* level of difficulty. *U_i,j_* represents the frequency of continuation *j* in the training texts with the *i-th* level of difficulty. $\sum_{i=1}^{n}U_{i,j}$ represents the sum of frequency of continuation *j* in the training texts with all levels of difficulty. *m* represents the number of the levels of difficulty.

According to Eqs 3 and 4, a resource table of the emerging tendency of continuations is constructed. In the table, the emerging tendency of a continuation in the training texts with specific level of difficulty, which is used in the texts with only one level of difficulty (single level of difficulty), is 1. Some unstable continuations influence the overall analysis of the tendency of text’s difficulty due to their both low frequency and usage in the training texts with only one level of difficulty. Therefore, it is necessary to process the above continuations, so as to improve the effectiveness of the measurement of text’s difficulty. The weight coefficient of the continuations with single level of difficulty and a ≥ 10 frequency is set as 1, and that of other continuations with single level of difficulty decreases with the decrease of their frequency. The weight coefficient scheme of continuations with single level of difficulty is shown in Table 8. The final weight coefficients of continuations with single level of difficulty are determined by comparing the measurement results of texts’ difficulty based on different sets of weight coefficients.

**Table 8:** Weight coefficient scheme of continuations with single level of difficulty.

Frequency	Weight coefficient
*f*> = 10	1
10>*f*> = 5	0.75
5>*f*> = 3	0.5
*f* = 2	0.25
*f* = 1	0.1

**Fig 3:**
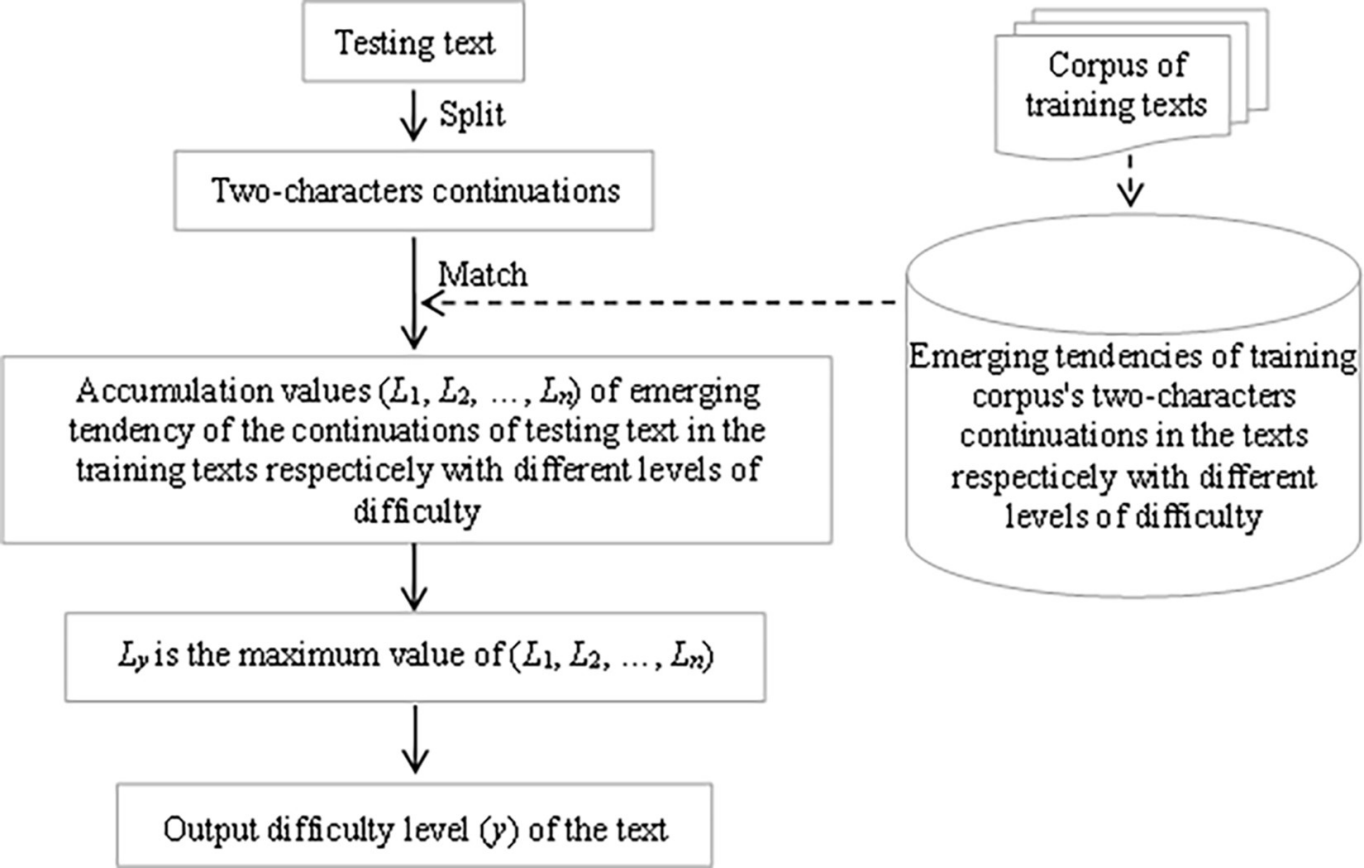
Working principle for measuring text’s difficulty based on emerging tendency of two-character continuations.

Method for measuring text’s difficulty based on emerging tendency of two-character continuation is described as follows, which can be seen in Fig 3. According to resource table of the emerging tendency of continuations, the accumulation values (*L*_1_, *L*_2_, …, *L*_n_) of emerging tendency of the continuations of testing text in the training texts respectively with different levels of difficulty are calculated. If *L*_*y*_ is the maximum value of (*L*_1_, *L*_2_, …, *L*_n_), the testing text’s level of difficulty is *y*.

Eq (5) shows the accumulation value of emerging tendency of the continuations of testing text in the training texts with the *i*-th level of difficulty.


$$L_{i}=\sum_{j=1}^{n}Q(i,j)$$ (5)

*i* represents a specific level. *j* represents a continuation in testing text. *n* represents the number of kinds of the continuations in testing text. *Q*(*i,j*) represents the emerging tendency of continuation *j* in the training texts with the *i-th* level of difficulty. *L_i_* represents the accumulation value of the emerging tendency of continuation *j* in the training texts with the *i-th* level of difficulty.

## Results and discussion

### Measurement results of text’s difficulty based on different methods

Tables 9–11 show the measurement results of testing texts’ difficulty with 6 levels, 3 levels and 2 levels difficulties based on different methods. As shown in the Tables 9–11, the method of measuring text difficulty based on new and stable continuations shows the highest overall effectiveness compared to other two methods. Its accuracy for measuring difficulty with 6 levels, 3 levels and 2 levels difficulties are 36.4%, 64.6% and 79.6%, respectively. The measurement method based on continuation index of character demonstrates better overall effectiveness. Its accuracy for measuring difficulty with 6 levels, 3 levels and 2 levels difficulties are 31.9%, 61.6% and 75.9%, respectively. In contrast, the measurement method based on emerging tendency of continuation shows the lowest overall effectiveness. Its accuracy for measuring difficulty with 6 levels, 3 levels and 2 levels difficulties are 35.2%, 55.1% and 75.2%, respectively.

**Table 9:** Measurement results of testing texts’ difficulty with 6 levels difficulty based on different methods.

Type Level	Continuation index of character	New and stable continuation	Emerging tendency of continuation
	6 levels accuracy	6 levels accuracy	6 levels accuracy
The 1st level	61.1%	63.9%	38.9%
The 2nd level	26.2%	33.3%	42.0%
The 3rd level	33.1%	44.1%	23.6%
The 4th level	24.5%	31.6%	19.4%
The 5th level	14.0%	26.3%	43.0%
The 6th level	56.5%	37.0%	47.8%
Total	**31.9%**	**36.4%**	**35.2%**

**Table 10:** Measurement results of testing texts’ difficulty with 3 levels difficulty based on different methods.

Type Level	Continuation index of character	New and stable continuation	Emerging tendency of continuation
The 1st level	69.1%	79.6%	69.1%
The 2nd level	44%	44.9%	30.7%
The 3rd level	71.8%	74.3%	70.9%
Total	**61.6%**	**64.6%**	**55.1%**

**Table 11:** Measurement results of testing texts’ difficulty with 2 levels difficulty based on different methods.

Type Level	Continuation index of character	New and stable continuation	Emerging tendency of continuation
The 1st level	68.5%	76.8%	76.1%
The 2nd level	82.9%	82.2%	74.3%
Total	**75.9%**	**79.6%**	**75.2%**

According to the dynamic change of the usage of continuations in the texts with different levels of difficulty, the measurement method of text’s difficulty based on new and stable continuations extracts the representatives of the new and stable continuations with single level of difficulty. The representatives in the training corpus are the key to measure the difficulty level of testing text. Therefore, the measurement method of text’s difficulty based on new and stable continuations has a high accuracy in the measurement of text’s difficulty. If more representatives are gained by expanding the number of texts in training corpus, the analysis of difficulty of all continuations in testing text can be refined and its measuring accuracy can be enhanced.

The method based on continuation index of character measures text’s difficulty by calculating the absolute distance between the average of continuation indexes of the characters in testing text and that of all texts with the same difficulty level in training corpus. Due to the analysis of the total difficulty of all characters used in testing text, the difficulties of all characters in testing text are comprehensively considered. Therefore, the measurement result of the testing text’s difficulty based on continuation index of character is better.

The difficulty of continuations obtained based on emerging tendency of continuation comprehensively reflects the numbers of continuations in different levels of difficulty. Meanwhile, according to different frequencies of the continuations only used in one level of difficulty, the various weight coefficients are assigned to those continuations. This can enhance the role of stable continuations in measuring text’s difficulty, while decreasing the role of unstable continuations. By comparing the accumulation values of the tendencies of all two-characters continuations of the testing text in the training texts respectively with different levels of difficulty, the level with the maximum in the accumulation values is determined as the testing text’s difficulty. However, for the continuations with a specific frequency, which is both unstable and only used in one level of difficulty, it is a complex problem for determining their weight coefficient. This leads to the non-ideal measurement result of text’s difficulty obtained by the above method.

### Comparison of difficulty measurement results between this study and previous research

The measurement results of the difficulty of composition testing texts in primary school using different methods are compared. As shown in Table 12, the measurement accuracy of texts’ difficulty with 6 levels difficulties based on Jiang’s method, is 35.5% [15], which is lower than that by this study. The research result shows that the measurement method of text’s difficulty based on new and stable continuations is effective, proving that the two-characters continuation can be used as a sensitive measurement unit of Chinese text’s difficulty.

**Table 12:** Measurement results of difficulty of composition testing texts in primary school using various methods.

Research work	Research object	Measurement method	Language characteristics	Accuracy(%)		
				6 levels	3 levels	2 levels
This study	Composition texts in primary school	*i*+1 algorithm	New and stable two-characters continuations	36.4	64.6	79.6
Jiang (2018)	Composition texts in primary school	Word embedding model, Convolutional neural network model	The acquiring, using and structuring difficulty of words	35.5	No research	No research

The measurement results of difficulty of Chinese textbook testing texts in primary school based on different methods are also compared. As can be seen in Table 13, the measurement accuracy of texts’ difficulty with 3 levels difficulty based on Wu’s method is 60.1% [16], which is lower than that using this study, indicating that the *i*+1 measurement method of language’s difficulty has universality.

**Table 13:** Measurement results of difficulty of Chinese textbook testing texts in primary school using various methods.

Research work	Research object	Measurement method	Language characteristics	Accuracy(%)		
				6 levels	3 levels	2 levels
This study	Chinese textbook texts in primary school	*i*+1 algorithm	New and stable two-characters continuations	44.9	66.2	84.7
Wu (2020)	Chinese textbook texts in primary school	Machine learning model	Character, word, sentence and chapter	No research	60.1	No research

## Conclusions

To enhance measurement accuracy of Chinese text’s difficulty, this paper investigates text’s difficulty based on a new perspective, which is two-characters continuation.

1. According to "continuation index of character", "new and stable two-characters continuation" and "emerging tendency of two-characters continuation", three various measurement methods are proposed to investigate the difficulty of text. It is found that compared to other two methods, the measurement method based on new and stable continuation is better in measuring text’s difficulty, and its accuracies for measuring text’s difficulty with 6 levels, 3 levels and 2 levels difficulties can reach 36.4%, 64.6% and 79.6%, respectively.
2. For composition and Chinese textbook testing texts in primary school, the measurement accuracies of text’s difficulty based on this study exceed those by Jiang and Wu’s methods. This demonstrates the effectiveness of measuring text’s difficulty based on new and stable continuations, highlighting two-character continuation as a sensitive unit for measuring Chinese text’s difficulty.

## Supporting information

S1 File S1 (Corpus of training texts of compositions in primary school). S2 (Corpus of testing texts of compositions in primary school). S3 (Corpus of testing texts of Chinese textbook in primary school). S4 (Minimal data set).
(RAR)
